# Clinical characteristics and surgical outcomes of acute acquired Comitant Esotropia

**DOI:** 10.1186/s12886-019-1182-2

**Published:** 2019-08-07

**Authors:** Chunyan Cai, Hongbin Dai, Yin Shen

**Affiliations:** 1 Eye center, Wuhan University Renmin Hospital, Wuhan, 430060 China; 2 Wuhan Aier Eye Hospital, Wuhan, 430063 China

**Keywords:** Acute acquired comitant esotropia, Strabismus surgery, Medial rectus insertion

## Abstract

**Background:**

To describe the clinical characteristics and the outcomes of strabismus surgery for acute acquired comitant esotropia (AACE).

**Methods:**

Medical records of 45 AACE patients were retrospectively analyzed. The insertion location of medial rectus was compared between the AACE patients and comitant exotropic patients. The location was also compared with those measured in other studies. Surgical outcome measurements included amount of deviation and level of binocularity at last follow-up.

**Results:**

The distance from medial rectus to limbus was shorter in AACE patients than in patients with comitant exotropia. The distance was also shorter in AACE patients than patients in other studies. Out of the 45 patients, 2 had neurological diseases. Neostigmine test was negative in all patients. The age at onset of AACE was 5–47 years (mean 19.1 ± 7.3 years), one patient was 5 years (2.2%), 20 patients were 11–17 years (44.5%) and the other 24 patients were 18–47 years old (53.3%). The mean cycloplegic refraction was − 4.1 ± 3.0 diopters (D) and 41 patients were myopic (91%). The angle of deviation was 40.5 ± 19.5 prism diopters (PD) at distance and 35.6 ± 19.9PD at near preoperatively. The angle was 0.8 ± 1.6 PD at distance and 0.7 ± 1.8 PD at near postoperatively. Diplopia resolved in patients who underwent strabismus surgery, with no recurrence during the follow-up period. Thirty patients had stereopsis postoperatively.

**Conclusions:**

AACE seems to occur mostly in older children and adults and myopes. The distance from the insertion to limbus of medial rectus was shorter in patients with AACE. Good results can be achieved by strabismus surgery.

## Background

Acute acquired comitant esotropia (AACE) is a relatively rare presentation of esotropia characterized by a sudden onset of comitant esotropia with diplopia, which often occurs in older children and adults [1–3]. There were a number of reports about the etiologies, clinical characteristics and treatment of AACE, but case series in most of the reports were limited and few studies focused on Chinese populations. AACE was considered to be related to accommodative spasm, myopia, hyperopia, intracranial diseases, excessive near work [1, 3, 4]. However, the etiologies remain unclear to date. Many studies focused on neurological etiologies, but no study has focused on the anatomy of medial and lateral rectus in AACE patients. A good binocular function may be potentially restored after appropriate treatment for AACE, but few studies describe the outcome of strabismus surgery. The aim of this study was to explore the etiologies and describe the clinical charateristics and the outcomes of strabismus surgery for AACE.

## Methods

Records of 45 patients diagnosed with AACE from November 2011 to July 2017 at the Eye Center, Renmin Hospital of Wuhan University were retrospectively analyzed in this study. Fifty comitant exotropic patients were also enrolled in this study. Our study was conducted in adherence to the tenets of Declaration of Helsinki and approved by the ethics committee of the hospital. The need for individual informed consent was waived by the ethics committee because of the retrospective nature of the study. Patients who met the criteria of AACE were included in this study: sudden onset of comitant esotropia (deviation difference was less than 5 prism diopters (PD) in all directions of gaze), normal eye movement, and accompanied diplopia. Patients with a history of eye surgery, paralysis, and accommodative strabismus (hyperopia ≥ + 2.00 D) were excluded.

A comprehensive medical history including general health status, family history, and previous ocular history was taken. All patients underwent ophthalmic and orthoptic examinations, cranial and orbital computed tomography (CT) or magnetic resonance imaging (MRI). Cycloplegic refraction was performed after administering 1% atropine ointment once daily for 7 days in patients younger than 12 years and 1% cyclopentolate eye drops every 5 min for three times for those > 12 years old. Spherical equivalents (SE) of refractive error were calculated using the algebraic sum of the dioptric powers of the sphere and half of the cylinder. Ocular motility was evaluated clinically. The deviation was measured using an alternate prism cover test in all nine gaze positions with and without refractive correction and was taken at near and distance fixation. The three grades of binocular function including simultaneous perception, fusion and stereopsis were evaluated by using a synoptophore (Inami& Co Ltd., Tokyo, Japan). Stereoacuity was assessed with the Titmus test card at 40 cm. The neostigmine test was used to exclude myasthenia gravis. Further, 44 patients underwent strabismus surgery. We chose to work on 1, 2 or 3 muscles or doing on the basis of angle of deviation both at distance and at near. We chose to work on 1 muscle when the angle was smaller than 20 PD, 2 muscles when the angle was 20PD to 60PD, 3 muscles when the angle was larger than 60PD. We chose to perform unilateral lateral rectus resection or medial rectus recession and lateral rectus resection when the angle of deviation was larger at distance than at near, unilateral or bilateral medial rectus recession when the angle was larger at near than at distance. The patient who was found with a mass in the pituitary underwent brain tumor surgery. The distance from the midpoint insertion of medial rectus to the sclerocorneal limbus was measured with a caliper before the muscle was cut and after the muscle was hooked in patients who underwent strabismus surgery [5, 6]. Deviation, stereopsis, and binocular function were remeasured during the follow-up period (1 week to 2 years). The insertion location of medial rectus was compared between the AACE patients and 50 exotropic patients. The location was also compared with those measured in other studies.

SPSS version 17.0 (SPSS Inc., Chicago, IL, USA) was used for statistical analysis. Shapiro-Wilk test was used to assess the normality of data. A *t* test was used to compare the deviation at near and distance fixations. We also used *t* test to compare the age, refractive error and insertion of medial rectus in AACE patients and exotropic patients. We used case number, mean and SD for *t* test comparison by using an online *t* test calculator as Lai [5] did in their study since we cannot get the raw data of Apt’s [7] and Lai’s [5] and Niyaz’s [6] studies (please also see http://www.graphpad.com/quickcalcs/ttest1.cfm). A *P* value of <0.05 was considered statistically significant.

## Results

The study included 45 AACE patients, 30 were males and 15 were females. The mean age was 21.6 ± 6.6 years and the mean age at onset of AACE was 5–47 years (mean 19.1 ± 7.3 years), one patient was 5 years (2.2%), 20 patients were 11–17 years old (44.5%) and the other 24 patients were 18–47 years old (53.3%). The time from onset to first visit was 1 month to 13 years (median 2 years). Moreover, 14 patients used smartphone or computer for more than 5 h per day before onset of AACE. None of the patients had a family history of strabismus, trauma, and occlusion of one eye. External and anterior segments were normal in all patients. The fundus examination was normal in all patients except one who had papilledema in both eyes and a mass in the pituitary region, who also complained of a headache. The neostigmine test was negative in all patients. One patient was found to have a mass in the pituitary region along with hydrocephalus on CT examination, and another patient was found to have demyelination on MRI examination without neurological signs. The other 43 patients were neuroradiologically negative.

The preoperative angle of deviation was 40.5 ± 19.5 PD (range, 15–90 PD) at distance and 35.6 ± 19.9PD (range, 10–90 PD) at near. The data of deviation both at near and distance fixation followed normal distribution (*P* = 0.123 and 0.186 respectively). No significant difference was found between far and near fixation (*P* = 0.098).

Baseline comparison of both the AACE group and the exotropic group were summarized in Table 1. The best-corrected visual acuity was better than 0 log minimum angle of resolution (logMAR) units (20/20) in both eyes in all patients. For the AACE group, one patient was emmetropic, 3 patients were mild hyperopic, and 41 patients were myopic. Further, 18 myopic patients had never or seldom worn glasses before onset of AACE, and the other 23 patients with myopia often wore glasses.

**Table 1:** Baseline comparison between the AACE group and exotropic group

	Number of cases	Age (year)	Refraction error (Mean ± SD)	Axial length (mm)
AACE group	45	21.6 ± 6.6	−4.1 ± 3.0	25.4 ± 1.4
exotropic group	50	21.3 ± 8.6	−3.7 ± 2.7	25.1 ± 1.4
*P*		0.862	0.49	0.34

Four patients underwent unilateral medial rectus recession, 4 bilateral medial rectus recession, 26 unilateral medial rectus recession and lateral rectus resection, 1 unilateral lateral rectus resection, and 9 three-muscle operation (bilateral medial rectus recession and lateral rectus resection). Diplopia resolved immediately after surgery in patients who underwent strabismus surgery, with no recurrence during the follow-up period. The postoperative angle of deviation was 0.8 ± 1.6 PD (range, 0–6 PD) at distance and 0.7 ± 1.8 PD (range, 0–6 PD) at near. Pre- and post-operative comparison was described in Table 2.

**Table 2:** Pre- and Post-operative comparison in patients with AACE

		Pre-operation	Post-operation
Angle of deviation (PD)	Distance	40.5 ± 19.5	0.8 ± 1.6
	Near	35.6 ± 19.9	0.7 ± 1.8
		Number of patients	
Synoptphone	simultaneous perception	25	34
	fusion	20	31
	stereopsis	11	22
Stereoacuity	> 800″	12	6
	60″ to 800″	18	9
	≤60″	15	30

The distance was measured in 43 patients, as one patient did not undergo strabismus surgery because of the mass in the pituitary and the other patient underwent only unilateral lateral rectus resection. The distance from the insertion of medial rectus to limbus was 4.8 ± 0.4 mm (range, 4–5 mm) in 43 AACE patients and 5.4 ± 0.4 mm (range, 4.5–6 mm) in 50 exotropic patients. The data of medial rectus insertion in AACE patients and exotropic patients followed normal distribution (*P* = 0.096 and 0.12 respectively). The distance was 5.3 ± 0.7 mm, 5.2 ± 0.9 mm, 5.6 ± 1.0 mm in Apt’s, Lai’s and Niyaz’s studies respectively. The difference was statistically significant between the AACE group and the exotropic group (*P* < 0.001). The distance was also statistically significant shorter in AACE patients in the present study than in Apt’s [7] and Lai’s [5] studies, when compared with the control subjects in these studies(*P* = 0.0001, *P*<0.0001, respectively.), but the difference was not statistically significant when compared with Niyaz’s study (*P* = 0.16) (Table 3).

**Table 3:** Comparison of distance from insertion to limbus of medial rectus in AACE patients and exotropic group in this study and other studies (mm)

	AACE group	Exotropic group	Lai [5]	Apt [7]	Niyaz [6]
Mean ± SD	4.8 ± 0.4	5.4 ± 0.4	5.2 ± 0.9	5.3 ± 0.7	5.6 ± 1.0
N	43	50	60	100	14
*P*		<0.001	<0.001	0.0001	0.16

## Discussion

AACE is characterized by an acute onset of esotropia with an equal angle of deviation in all fields of gaze and accompanied diplopia and was classified into three types by Burian and Miller [3, 1) Swan type: acute comitant esotropia due to monocular occlusion or loss of vision in one eye; (2) Burian–Franceschetti type: associated with physical or psychological stress, low hyperopia, and minimal accommodative element; (3) Bielschowsky type: esotropia in patients with myopia of − 5.00 D or higher and the angle of deviation larger for distance than for near, or esotropia occurring only at distance but fusion at near, with no evidence of lateral rectus paralysis [8]. In the present study, no patient had a history of monocular occlusion or visual loss in one eye, 4 patients were mild hyperopic and the other 41 were myopic. According to the classification, 4 patients belonged to the Burian–Franceschetti type, 41 belonged to the Bielschowsky type, but no patient belonged to the Swan type. Although the angle was a little larger at far than at near in this study, the difference was not statistically significant. The study by Chen et al. [9] on 47 patients also reported no patient belonging to the Swan type of AACE. Bielschowsky et al. [8] claimed that uncorrected myopia was the etiology of AACE. The underlying mechanism was that uncorrected myopia might lead to excessive near work, resulting in the imbalance between the convergence and divergence forces of the eyes and subsequent development of increased tonus of the medial rectus muscles, leading to esotropia. In the present study, 23 out of 41 myopic patients of the Bielschowsky type wore glasses. Chen et al. [9] also found that most myopic patients of the Bielschowsky type wore glasses in their study and concluded that uncorrected myopia could not be the etiology of the Bielschowsky type. Thus, whether uncorrected myopia was the etiology of this subtype of AACE was not confirmed.

Recently, Lee et al. [10] reported that AACE was associated with excessive smartphone use. The mechanism was similar to that proposed by Bielschowsky [8]. In the present study, 14 patients had a history of smartphone or computer use for more than 5 h per day.

The mechanism of AACE is still not clear. Neurological diseases are considered to be associated with this type of strabismus. The morbidity was from 0 to 10% in previous studies of different sizes of samples [1, 2, 9, 11]. In the present study, only two patients presented with intracranial diseases (4.4%). Thus, it was believed that the etiology was benign in most patients with AACE. Since the majority of patients with AACE have no obvious underlying neurological causes, the question is whether every patient with AACE deserves a neurological evaluation. It is still controversial. Some researchers agreed that AACE is benign and might be with no risk of associated intracranial pathology [12]. However, in this study, although all the patients were comitant, two patients were neuroradiologically positive. William et al. [13] claimed that comitancy in AACE did not rule out the possibility of an underlying serious neurological condition. There are many exceptions because AACE can be the first or sole sign of intracranial diseases [14, 15]. Moreover, the other researchers held different opinions. Some researchers suggested that neurological pathology should be taken into consideration when neurological signs existed [1, 9, 11, 16, 17]. But Clark et al [2] recommended that all patients with AACE, especially type II, should undergo complete neurological and ophthalmological examinations, although the results of neurological examination were negative in their study of 10 patients. Hoyt et al. [16] suggested that neurological cause was not a concern when associated with a history of previous strabismus, occlusion therapy, monocular visual loss, or myopia. Since the patient with AACE who was found to have mass in the pituitary region was myopic in the present study, it is not presumed that previous myopia can rule out the possibility of neurological condition. Thus, it is thought that neurological pathology should be taken into consideration for each patient with AACE.

It is important to have the knowledge of the anatomic characteristics of extraocular muscles. The muscle insertion and its anatomy are important for understanding the physiology of eye movements. However, available anatomic studies on insertions of extraocular muscles especially in vivo measurements are very few [5, 6] and no study on AACE has focused on anatomic characteristics of extraocular muscles. Among all the extraocular muscles, Lai et al. [5] found the insertion of the medial rectus seemed to be the most reliable and invariant of the rectus muscles in all patients regardless of their ethnic background. It is well known that the distance from the insertion location of medial rectus to limbus is about 5.3 mm in Western populations [7]. Athavale et al. [18] and Surawatsatien et al. [19] found the distance of insertion of medial rectus to the limbus was 7.3 mm in Indian population and 5.7 mm in Thai population respectively from the measurement of cadavers. Lai et al. [5] and Niyaz et al. [6] measured the distance of medial rectus by a caliper in the esotropic and exotropic patients who underwent strabismus surgery and in the control group when a buckling surgery for retinal detachment was performed. Lai et al. [5] found the distance from the insertion location to the limbus of medial rectus was 5.2 mm in the control group and 5.3 mm in the strabismus group in Taiwanese (Han Chinese) population. Niyaz et al. [6] found the distance for the medial rectus was 5.7 mm in esotropic group, 6.0 mm in exotropic group and 5.6 mm in the control group respectively in Turkish population. Lai et al. [5] and Niyaz et al. [6] found medial rectus insertion was not statistically different in esotropia, exotropia, and control patients. But in this study, the distance of medial rectus insertion was shorter in AACE patients than in exotropic patients. The distance of medial rectus was also shorter than that measured in control group in Lai’s study [5]. It is thus assumed that the shorter distance of medial rectus may lead to the strengthening of convergence tonus, resulting in the imbalance between convergence and divergence and subsequent esotropia. However, the abnormal insertion of medial rectus in AACE patients was not described in previous studies. We also found that the distance was significantly shorter in AACE patients than in Western populations [7], which was consistent with Lai et al. [5]. The difference between Chinese and Western populations may result from the different ethnic background, and the difference may affect surgical outcome as Lai et al. [5] suggested. Good binocular function may be potentially restored after appropriate treatment because AACE often occurs after binocular vision is well developed. In the present study, diplopia resolved in all patients who underwent strabismus surgery, and the majority of patients regained binocular function. No recurrence of AACE was found during the follow-up period. Thus, it is suggested that surgery is a good choice for patients with AACE.

This study had some limitations. First, the sample size was not big enough and hence other etiologies were not found. A larger, multicenter study can provide more conclusions about the etiologies and outcome of strabismus surgery. Second, the duration of follow-up was not long enough, and therefore long-term stability of eye alignment after strabismus surgery is needed to provide more answers. Third, because of the retrospective nature of our study, data were limited to what was available in the patients records, we only measured the distance from insertion to limbus of medial rectus, the data of the distance of lateral rectus and the width of medial and lateral rectus cannot be obtained. Niyaz et al. [6] found that the width of medial rectus was related to the size of deviation in esotropia. Therefore, whether extraocular rectus width affected the size of deviation was not explored, demanding further studies. In addition, limited to the available data, we cannot compare the distance of medial rectus with control subjects with normal age and refractive error matched individuals, we can only compare the distance of medial rectus with control subjects in other studies. Further comparison between AACE patients and control and other types of esotropia are recommended.

## Conclusions

AACE seems to occur mostly in older children and adults and myopes. The distance from the insertion of medial rectus to limbus was shorter in patients with AACE. The possibility of a serious, underlying neurological condition should be considered in all patients, although most patients are neurologically negative. Good motor and sensory outcomes can be achieved in patients without neurological diseases via appropriate strabismus surgery.

## Data Availability

The data are available from the corresponding author upon reasonable request.

## Abbreviations

AACE: Acute acquired comitant esotropia
CT: Computed tomography
MRI: Magnetic resonance imaging
PD: Prism diopters
SE: Spherical equivalents
